# Learning effect of online versus onsite education in health and medical scholarship – protocol for a cluster randomized trial

**DOI:** 10.1186/s12909-024-05915-z

**Published:** 2024-08-26

**Authors:** Rie Raffing, Lars Konge, Hanne Tønnesen

**Affiliations:** 1 WHO Collaborating Centre (DEN-62), Clinical Health Promotion Centre, The Parker Institute, Bispebjerg & Frederiksberg Hospital, University of Copenhagen, Copenhagen, 2400 Denmark; 2 Copenhagen Academy for Medical Education and Simulation (CAMES), Centre for HR and Education, The Capital Region of Denmark, Copenhagen, 2100 Denmark

**Keywords:** Teaching, Learning, Preference, Motivation, Self-efficacy, Achievements, Online, Onsite, Health and Medical education, Randomized

## Abstract

**Background:**

The disruption of health and medical education by the COVID-19 pandemic made educators question the effect of online setting on students’ learning, motivation, self-efficacy and preference. In light of the health care staff shortage online scalable education seemed relevant. Reviews on the effect of online medical education called for high quality RCTs, which are increasingly relevant with rapid technological development and widespread adaption of online learning in universities. The objective of this trial is to compare standardized and feasible outcomes of an online and an onsite setting of a research course regarding the efficacy for PhD students within health and medical sciences: Primarily on learning of research methodology and secondly on preference, motivation, self-efficacy on short term and academic achievements on long term. Based on the authors experience with conducting courses during the pandemic, the hypothesis is that student preferred onsite setting is different to online setting.

**Methods:**

Cluster randomized trial with two parallel groups. Two PhD research training courses at the University of Copenhagen are randomized to online (Zoom) or onsite (The Parker Institute, Denmark) setting. Enrolled students are invited to participate in the study. Primary outcome is short term learning. Secondary outcomes are short term preference, motivation, self-efficacy, and long-term academic achievements. Standardized, reproducible and feasible outcomes will be measured by tailor made multiple choice questionnaires, evaluation survey, frequently used Intrinsic Motivation Inventory, Single Item Self-Efficacy Question, and Google Scholar publication data. Sample size is calculated to 20 clusters and courses are randomized by a computer random number generator. Statistical analyses will be performed blinded by an external statistical expert.

**Discussion:**

Primary outcome and secondary significant outcomes will be compared and contrasted with relevant literature. Limitations include geographical setting; bias include lack of blinding and strengths are robust assessment methods in a well-established conceptual framework. Generalizability to PhD education in other disciplines is high. Results of this study will both have implications for students and educators involved in research training courses in health and medical education and for the patients who ultimately benefits from this training.

**Trial registration:**

Retrospectively registered at ClinicalTrials.gov: NCT05736627. SPIRIT guidelines are followed.

**Supplementary Information:**

The online version contains supplementary material available at 10.1186/s12909-024-05915-z.

## Background

Medical education was utterly disrupted for two years by the COVID-19 pandemic. In the midst of rearranging courses and adapting to online platforms we, with lecturers and course managers around the globe, wondered what the conversion to online setting did to students’ learning, motivation and self-efficacy [1–3]. What the long-term consequences would be [4] and if scalable online medical education should play a greater role in the future [5] seemed relevant and appealing questions in a time when health care professionals are in demand. Our experience of performing research training during the pandemic was that although PhD students were grateful for courses being available, they found it difficult to concentrate related to the long screen hours. We sensed that most students preferred an onsite setting and perceived online courses a temporary and inferior necessity. The question is if this impacted their learning?

Since the common use of the internet in medical education, systematic reviews have sought to answer if there is a difference in learning effect when taught online compared to onsite. Although authors conclude that online learning may be equivalent to onsite in effect, they agree that studies are heterogeneous and small [6, 7], with low quality of the evidence [8, 9]. They therefore call for more robust and adequately powered high-quality RCTs to confirm their findings and suggest that students’ preferences in online learning should be investigated [7–9].

This uncovers two knowledge gaps: I) High-quality RCTs on online versus onsite learning in health and medical education and II) Studies on students’ preferences in online learning.

Recently solid RCTs have been performed on the topic of web-based theoretical learning of research methods among health professionals [10, 11]. However, these studies are on asynchronous courses among medical or master students with short term outcomes.

This uncovers three additional knowledge gaps: III) Studies on synchronous online learning IV) among PhD students of health and medical education V) with long term measurement of outcomes.

The rapid technological development including artificial intelligence (AI) and widespread adaption as well as application of online learning forced by the pandemic, has made online learning well-established. It represents high resolution live synchronic settings which is available on a variety of platforms with integrated AI and options for interaction with and among students, chat and break out rooms, and exterior digital tools for teachers [12–14]. Thus, investigating online learning today may be quite different than before the pandemic. On one hand, it could seem plausible that this technological development would make a difference in favour of online learning which could not be found in previous reviews of the evidence. On the other hand, the personal face-to-face interaction during onsite learning may still be more beneficial for the learning process and combined with our experience of students finding it difficult to concentrate when online during the pandemic we hypothesize that outcomes of the onsite setting are different from the online setting.

To support a robust study, we design it as a cluster randomized trial. Moreover, we use the well-established and widely used Kirkpatrick’s conceptual framework for evaluating learning as a lens to assess our outcomes [15]. Thus, to fill the above-mentioned knowledge gaps, the objective of this trial is to compare a synchronous online and an in-person onsite setting of a research course regarding the efficacy for PhD students within the health and medical sciences:

- Primarily on theoretical learning of research methodology and
- Secondly on
  - ◦ Preference, motivation, self-efficacy on short term
  - ◦ Academic achievements on long term

## Methods

### Trial design

**Fig. 1:**
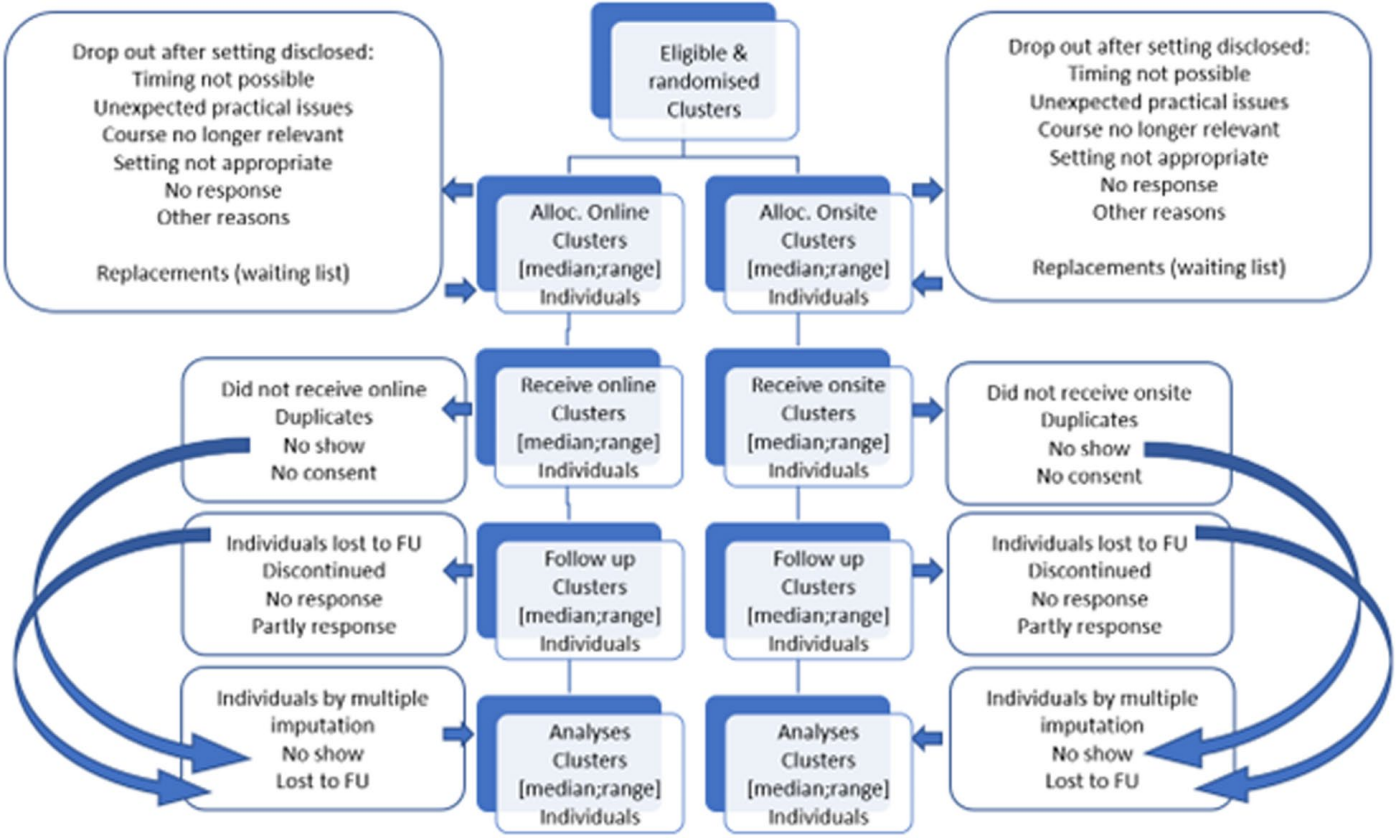
Consort flow diagram

This study protocol covers synchronous online and in-person onsite setting of research courses testing the efficacy for PhD students. It is a two parallel arms cluster randomized trial (Fig. 1).

The study measures baseline and post intervention. Baseline variables and knowledge scores are obtained at the first day of the course, post intervention measurement is obtained the last day of the course (short term) and monthly for 24 months (long term).

Randomization is stratified giving 1:1 allocation ratio of the courses. As the number of participants within each course might differ, the allocation ratio of participants in the study will not fully be equal and 1:1 balanced.

### Study setting

The study site is The Parker Institute at Bispebjerg and Frederiksberg Hospital, University of Copenhagen, Denmark. From here the courses are organized and run online and onsite. The course programs and time schedules, the learning objective, the course management, the lecturers, and the delivery are identical in the two settings. The teachers use the same introductory presentations followed by training in break out groups, feed-back and discussions. For the online group, the setting is organized as meetings in the online collaboration tool Zoom® [16] using the basic available technicalities such as screen sharing, chat function for comments, and breakout rooms and other basics digital tools if preferred. The online version of the course is synchronous with live education and interaction. For the onsite group, the setting is the physical classroom at the learning facilities at the Parker Institute. Coffee and tea as well as simple sandwiches and bottles of water, which facilitate sociality, are available at the onsite setting. The participants in the online setting must get their food and drink by themselves, but online sociality is made possible by not closing down the online room during the breaks. The research methodology courses included in the study are “Practical Course in Systematic Review Technique in Clinical Research”, (see course programme in appendix 1) and “Getting started: Writing your first manuscript for publication” [17] (see course programme in appendix 2). The two courses both have 12 seats and last either three or three and a half days resulting in 2.2 and 2.6 ECTS credits, respectively. They are offered by the PhD School of the Faculty of Health and Medical Sciences, University of Copenhagen. Both courses are available and covered by the annual tuition fee for all PhD students enrolled at a Danish university.

### Eligibility criteria

Inclusion criteria for participants: All PhD students enrolled on the PhD courses participate after informed consent: “Practical Course in Systematic Review Technique in Clinical Research” and “Getting started: Writing your first manuscript for publication” at the PhD School of the Faculty of Health and Medical Sciences, University of Copenhagen, Denmark.

Exclusion criteria for participants: Declining to participate and withdrawal of informed consent.

### Informed consent

The PhD students at the PhD School at the Faculty of Health Sciences, University of Copenhagen participate after informed consent, taken by the daily project leader, allowing evaluation data from the course to be used after pseudo-anonymization in the project. They are informed in a welcome letter approximately three weeks prior to the course and again in the introduction the first course day. They register their consent on the first course day (Appendix 3). Declining to participate in the project does not influence their participation in the course.

### Interventions

Online course settings will be compared to onsite course settings. We test if the onsite setting is different to online. Online learning is increasing but onsite learning is still the preferred educational setting in a medical context. In this case onsite learning represents “usual care”. The online course setting is meetings in Zoom using the technicalities available such as chat and breakout rooms. The onsite setting is the learning facilities, at the Parker Institute, Bispebjerg and Frederiksberg Hospital, The Capital Region, University of Copenhagen, Denmark.

The course settings are not expected to harm the participants, but should a request be made to discontinue the course or change setting this will be met, and the participant taken out of the study. Course participants are allowed to take part in relevant concomitant courses or other interventions during the trial.

### Strategies to improve adherence to interventions

Course participants are motivated to complete the course irrespectively of the setting because it bears ECTS-points for their PhD education and adds to the mandatory number of ECTS-points. Thus, we expect adherence to be the same in both groups. However, we monitor their presence in the course and allocate time during class for testing the short-term outcomes ( motivation, self-efficacy, preference and learning). We encourage and, if necessary, repeatedly remind them to register with Google Scholar for our testing of the long-term outcome (academic achievement).

### Outcomes

**Fig. 2:**
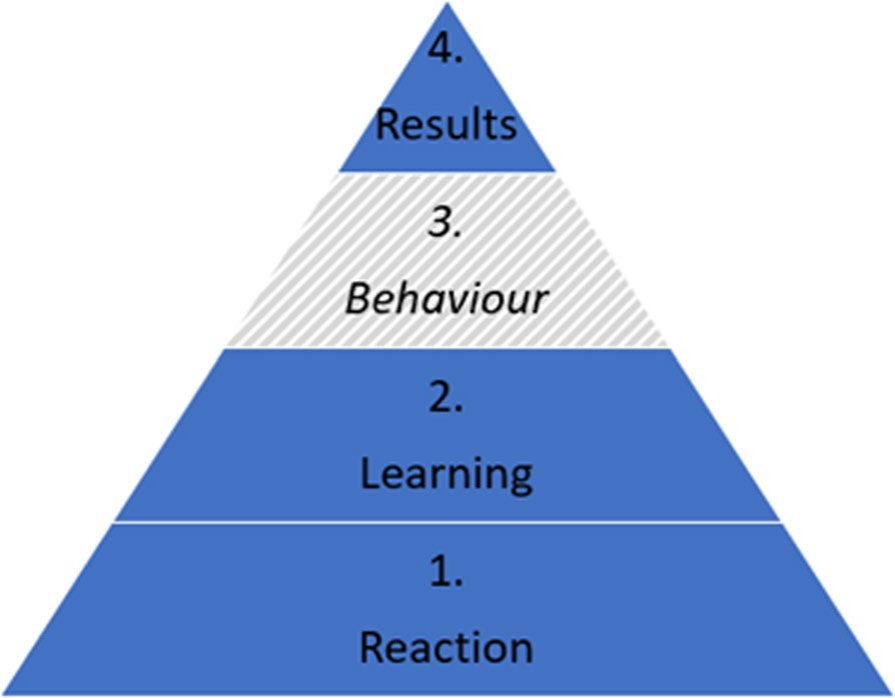
The Kirkpatrick model

Outcomes are related to the Kirkpatrick model for evaluating learning (Fig. 2) which divides outcomes into four different levels; *Reaction* which includes for example motivation, self-efficacy and preferences, *Learning* which includes knowledge acquisition, *Behaviour* for practical application of skills when back at the job (not included in our outcomes), and *Results* for impact for end-users which includes for example academic achievements in the form of scientific articles [18–20].

### Primary outcome

The primary outcome is short term learning (Kirkpatrick level 2).

Learning is assessed by a Multiple-Choice Questionnaire (MCQ) developed prior to the RCT specifically for this setting (Appendix 4). First the lecturers of the two courses were contacted and asked to provide five multiple choice questions presented as a stem with three answer options; one correct answer and two distractors. The questions should be related to core elements of their teaching under the heading of research training. The questions were set up to test the cognition of the students at the levels of "Knows" or "Knows how" according to Miller's Pyramid of Competence and not their behaviour [21]. Six of the course lecturers responded and out of this material all the questions which covered curriculum of both courses were selected. It was tested on 10 PhD students and within the lecturer group, revised after an item analysis and English language revised. The MCQ ended up containing 25 questions. The MCQ is filled in at baseline and repeated at the end of the course. The primary outcomes based on the MCQ is estimated as the score of learning calculated as number of correct answers out of 25 after the course. A decrease of points of the MCQ in the intervention groups denotes a deterioration of learning. In the MCQ the minimum score is 0 and 25 is maximum, where 19 indicates passing the course.

Furthermore, as secondary outcome, this outcome measurement will be categorized as binary outcome to determine passed/failed of the course defined by 75% (19/25) correct answers.

The learning score will be computed on group and individual level and compared regarding continued outcomes by the Mann–Whitney test comparing the learning score of the online and onsite groups. Regarding the binomial outcome of learning (passed/failed) data will be analysed by the Fisher’s exact test on an intention-to-treat basis between the online and onsite. The results will be presented as median and range and as mean and standard deviations, for possible future use in meta-analyses.

### Secondary outcomes

Motivation assessment post course: Motivation level is measured by the Intrinsic Motivation Inventory (IMI) Scale [22] (Appendix 5). The IMI items were randomized by random.org on the 4th of August 2022. It contains 12 items to be assessed by the students on a 7-point Likert scale where 1 is “Not at all true”, 4 is “Somewhat true” and 7 is “Very true”. The motivation score will be computed on group and individual level and will then be tested by the Mann–Whitney of the online and onsite group.

Self-efficacy assessment post course: Self-efficacy level is measured by a single-item measure developed and validated by Williams and Smith [23] (Appendix 6). It is assessed by the students on a scale from 1–10 where 1 is “Strongly disagree” and 10 is “Strongly agree”. The self-efficacy score will be computed on group and individual level and tested by a Mann–Whitney test to compare the self-efficacy score of the online and onsite group.

Preference assessment post course: Preference is measured as part of the general course satisfaction evaluation with the question “If you had the option to choose, which form would you prefer this course to have?” with the options “onsite form” and “online form”.

Academic achievement assessment is based on 24 monthly measurements post course of number of publications, number of citations, h-index, i10-index. This data is collected through the Google Scholar Profiles [24] of the students as this database covers most scientific journals. Associations between onsite/online and long-term academic will be examined with Kaplan Meyer and log rank test with a significance level of 0.05.

### Participant timeline

Enrolment for the course at the Faculty of Health Sciences, University of Copenhagen, Denmark, becomes available when it is published in the course catalogue. In the course description the course location is “To be announced”. Approximately 3–4 weeks before the course begins, the participant list is finalized, and students receive a welcome letter containing course details, including their allocation to either the online or onsite setting. On the first day of the course, oral information is provided, and participants provide informed consent, baseline variables, and base line knowledge scores.

The last day of scheduled activities the following scores are collected, knowledge, motivation, self-efficacy, setting preference, and academic achievement. To track students' long term academic achievements, follow-ups are conducted monthly for a period of 24 months, with assessments occurring within one week of the last course day (Table 1).

**Table 1:**
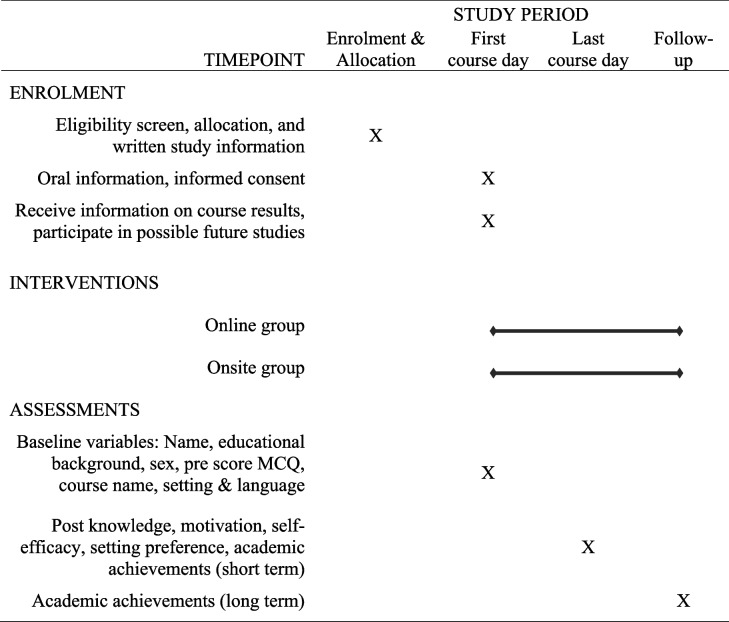
Schedule of enrolment, interventions, and assessments

### Sample size

The power calculation is based on the main outcome, theoretical learning on short term. For the sample size determination, we considered 12 available seats for participants in each course. To achieve statistical power, we aimed for 8 clusters in both online and onsite arms (in total 16 clusters) to detect an increase in learning outcome of 20% (learning outcome increase of 5 points). We considered an intraclass correlation coefficient of 0.02, a standard deviation of 10, a power of 80%, and a two-sided alpha level of 5%. The Allocation Ratio was set at 1, implying an equal number of subjects in both online and onsite group.

Considering a dropout up to 2 students per course, equivalent to 17%, we determined that a total of 112 participants would be needed. This calculation factored in 10 clusters of 12 participants per study arm, which we deemed sufficient to assess any changes in learning outcome.

The sample size was estimated using the function n4means from the R package CRTSize [25].

### Recruitment

Participants are PhD students enrolled in 10 courses of “Practical Course in Systematic Review Technique in Clinical Research” and 10 courses of “Getting started: Writing your first manuscript for publication” at the PhD School of the Faculty of Health Sciences, University of Copenhagen, Denmark.

### Assignment of interventions: allocation

Randomization will be performed on course-level. The courses are randomized by a computer random number generator [26]. To get a balanced randomization per year, 2 sets with 2 unique random integers in each, taken from the 1–4 range is requested.

The setting is not included in the course catalogue of the PhD School and thus allocation to online or onsite is concealed until 3–4 weeks before course commencement when a welcome letter with course information including allocation to online or onsite setting is distributed to the students. The lecturers are also informed of the course setting at this time point. If students withdraw from the course after being informed of the setting, a letter is sent to them enquiring of the reason for withdrawal and reason is recorded (Appendix 7).

The allocation sequence is generated by a computer random number generator (random.org). The participants and the lecturers sign up for the course without knowing the course setting (online or onsite) until 3–4 weeks before the course.

### Assignment of interventions: blinding

Due to the nature of the study, it is not possible to blind trial participants or lecturers. The outcomes are reported by the participants directly in an online form, thus being blinded for the outcome assessor, but not for the individual participant. The data collection for the long-term follow-up regarding academic achievements is conducted without blinding. However, the external researcher analysing the data will be blinded.

### Data collection and management

Data will be collected by the project leader (Table 1). Baseline variables and post course knowledge, motivation, and self-efficacy are self-reported through questionnaires in SurveyXact® [27]. Academic achievements are collected through Google Scholar profiles of the participants.

Given that we are using participant assessments and evaluations for research purposes, all data collection – except for monthly follow-up of academic achievements after the course – takes place either in the immediate beginning or ending of the course and therefore we expect participant retention to be high.

Data will be downloaded from SurveyXact and stored in a locked and logged drive on a computer belonging to the Capital Region of Denmark. Only the project leader has access to the data.

This project conduct is following the Danish Data Protection Agency guidelines of the European GDPR throughout the trial. Following the end of the trial, data will be stored at the Danish National Data Archive which fulfil Danish and European guidelines for data protection and management.

### Statistical methods

Data is anonymized and blinded before the analyses. Analyses are performed by a researcher not otherwise involved in the inclusion or randomization, data collection or handling. All statistical tests will be testing the null hypotheses assuming the two arms of the trial being equal based on corresponding estimates. Analysis of primary outcome on short-term learning will be started once all data has been collected for all individuals in the last included course. Analyses of long-term academic achievement will be started at end of follow-up.

Baseline characteristics including both course- and individual level information will be presented. Table 2 presents the available data on baseline.

**Table 2:** Baseline patient characteristics of participants

VARIABLES AT COURSE LEVEL		
	Name	Practical Course in Systematic Review Technique in Clinical Research; Getting started: Writing your first manuscript for publication
	Setting	Online; Onsite
	Language	Danish; English
VARIABLES AT INDIVIDUAL LEVEL		
	Sex	Male; Female
	Education prior to PhD study	MD; MSc Public Health; University College + MSc; MSc Other
	Pre-test knowledge scores	0-25

We will use multivariate analysis for identification of the most important predictors (motivation, self-efficacy, sex, educational background, and knowledge) for best effect on short and long term. The results will be presented as risk ratio (RR) with 95% confidence interval (CI). The results will be considered significant if CI does not include the value one.

All data processing and analyses were conducted using R statistical software version 4.1.0, 2021–05-18 (R Foundation for Statistical Computing, Vienna, Austria).

If possible, all analysis will be performed for “Practical Course in Systematic Review Technique in Clinical Research” and for “Getting started: Writing your first manuscript for publication” separately.

Primary analyses will be handled with the intention-to-treat approach. The analyses will include all individuals with valid data regardless of they did attend the complete course. Missing data will be handled with multiple imputation [28]*.*

Upon reasonable request, public assess will be granted to protocol, datasets analysed during the current study, and statistical code Table 3.

**Table 3:** Outcome framework, measurements and analyses

**Kirkpatrick**	**Term**	**Outcome**	**Tool**	**Measurement**	**Analysis**
Primary outcome					
Level 2 (Learning)	Short	Knowledge	Tested MCQ	25 questions with 3 integers	Mann–Whitney
Level 2 (Learning)	Short	Knowledge Passed	Tested MCQ	Binary	Fisher’s exact
Secondary outcomes					
Level 1 (Reaction)	Short	Motivation	Intrinsic Motivation Inventory	12 questions on 7-point Likert scale	Mann–Whitney
Level 1 (Reaction)	Short	Self-efficacy	Williams and Smith	1 question on 10 point Likert scale	Mann–Whitney
Level 1 (Reaction)	Short	Preference	Survey	Binary	Fisher’s exact
Level 4 (Results)	Long	Academic achievements	Google Scholar	Publications, citations, h-index, and i10-index	Kaplan Meyer and log rank test

### Oversight, monitoring, and adverse events

This project is coordinated in collaboration between the WHO CC (DEN-62) at the Parker Institute, CAMES, and the PhD School at the Faculty of Health and Medical Sciences, University of Copenhagen. The project leader runs the day-to-day support of the trial. The steering committee of the trial includes principal investigators from WHO CC (DEN-62) and CAMES and the project leader and meets approximately three times a year.

Data monitoring is done on a daily basis by the project leader and controlled by an external independent researcher.

An adverse event is “a harmful and negative outcome that happens when a patient has been provided with medical care” [29]. Since this trial does not involve patients in medical care, we do not expect adverse events. If participants decline taking part in the course after receiving the information of the course setting, information on reason for declining is sought obtained. If the reason is the setting this can be considered an unintended effect. Information of unintended effects of the online setting (the intervention) will be recorded. Participants are encouraged to contact the project leader with any response to the course in general both during and after the course.

The trial description has been sent to the Scientific Ethical Committee of the Capital Region of Denmark (VEK) (21041907), which assessed it as not necessary to notify and that it could proceed without permission from VEK according to the Danish law and regulation of scientific research. The trial is registered with the Danish Data Protection Agency (Privacy) (P-2022–158). Important protocol modification will be communicated to relevant parties as well as VEK, the Joint Regional Information Security and Clinicaltrials.gov within an as short timeframe as possible.

### Dissemination plans

The results (positive, negative, or inconclusive) will be disseminated in educational, scientific, and clinical fora, in international scientific peer-reviewed journals, and clinicaltrials.gov will be updated upon completion of the trial. After scientific publication, the results will be disseminated to the public by the press, social media including the website of the hospital and other organizations – as well as internationally via WHO CC (DEN-62) at the Parker Institute and WHO Europe.

All authors will fulfil the ICMJE recommendations for authorship, and RR will be first author of the articles as a part of her PhD dissertation. Contributors who do not fulfil these recommendations will be offered acknowledgement in the article.

## Discussion

This cluster randomized trial investigates if an onsite setting of a research course for PhD students within the health and medical sciences is different from an online setting. The outcomes measured are learning of research methodology (primary), preference, motivation, and self-efficacy (secondary) on short term and academic achievements (secondary) on long term.

The results of this study will be discussed as follows:

- Discussion of primary outcome

Primary outcome will be compared and contrasted with similar studies including recent RCTs and mixed-method studies on online and onsite research methodology courses within health and medical education [10, 11, 30] and for inspiration outside the field [31, 32]: Tokalic finds similar outcomes for online and onsite, Martinic finds that the web-based educational intervention improves knowledge, Cheung concludes that the evidence is insufficient to say that the two modes have different learning outcomes, Kofoed finds online setting to have negative impact on learning and Rahimi-Ardabili presents positive self-reported student knowledge. These conflicting results will be discussed in the context of the result on the learning outcome of this study. The literature may change if more relevant studies are published.

- Discussion of secondary outcomes

Secondary significant outcomes are compared and contrasted with similar studies.

### Limitations, generalizability, bias and strengths

It is a limitation to this study, that an onsite curriculum for a full day is delivered identically online, as this may favour the onsite course due to screen fatigue [33]. At the same time, it is also a strength that the time schedules are similar in both settings. The offer of coffee, tea, water, and a plain sandwich in the onsite course may better facilitate the possibility for socializing. Another limitation is that the study is performed in Denmark within a specific educational culture, with institutional policies and resources which might affect the outcome and limit generalization to other geographical settings. However, international students are welcome in the class.

In educational interventions it is generally difficult to blind participants and this inherent limitation also applies to this trial [11]. Thus, the participants are not blinded to their assigned intervention, and neither are the lecturers in the courses. However, the external statistical expert will be blinded when doing the analyses.

We chose to compare in-person onsite setting with a synchronous online setting. Therefore, the online setting cannot be expected to generalize to asynchronous online setting. Asynchronous delivery has in some cases showed positive results and it might be because students could go back and forth through the modules in the interface without time limit [11].

We will report on all the outcomes defined prior to conducting the study to avoid selective reporting bias.

It is a strength of the study that it seeks to report outcomes within the 1, 2 and 4 levels of the Kirkpatrick conceptual framework, and not solely on level 1. It is also a strength that the study is cluster randomized which will reduce “infections” between the two settings and has an adequate power calculated sample size and looks for a relevant educational difference of 20% between the online and onsite setting.

### Perspectives with implications for practice

The results of this study may have implications for the students for which educational setting they choose. Learning and preference results has implications for lecturers, course managers and curriculum developers which setting they should plan for the health and medical education. It may also be of inspiration for teaching and training in other disciplines. From a societal perspective it also has implications because we will know the effect and preferences of online learning in case of a future lock down.

Future research could investigate academic achievements in online and onsite research training on the long run (Kirkpatrick 4); the effect of blended learning versus online or onsite (Kirkpatrick 2); lecturers’ preferences for online and onsite setting within health and medical education (Kirkpatrick 1) and resource use in synchronous and asynchronous online learning (Kirkpatrick 5).

## Trial status

This trial collected pilot data from August to September 2021 and opened for inclusion in January 2022. Completion of recruitment is expected in April 2024 and long-term follow-up in April 2026. Protocol version number 1 03.06.2022 with amendments 30.11.2023.

### Supplementary Information

Supplementary Material 1.

Supplementary Material 2.

Supplementary Material 3.

Supplementary Material 4.

Supplementary Material 5.

Supplementary Material 6.

Supplementary Material 7.

## Data Availability

The project leader will have access to the final trial dataset which will be available upon reasonable request. Exception to this is the qualitative raw data that might contain information leading to personal identification.

## Abbreviations

AI: Artificial Intelligence
CAMES: Copenhagen academy for medical education and simulation
CI: Confidence interval
COVID: Coronavirus disease
ECTS: European credit transfer and accumulation system
ICMJE: International committee of medical journal editors
IMI: Intrinsic motivation inventory
MCQ: Multiple choice questionnaire
MD: Doctor of medicine
MSc: Masters of sciences
RCT: Randomized controlled trial
VEK: Scientific ethical committee of the Capital Region of Denmark
WHO CC: WHO Collaborating centre for evidence-based clinical health promotion
