# Supplementary material for: Learning effect of online versus onsite education in health and medical scholarship – protocol for a cluster randomized trial
Source: BMC Med Educ. 2024 Aug 26;24:927. doi: 10.1186/s12909-024-05915-z (PMC11348670; doi:10.1186/s12909-024-05915-z)
Supplement: Supplementary file 1 — Supplementary Material 1. [file 12909_2024_5915_MOESM1_ESM.pdf]

Practical Course in Systematic Review Technique in Clinical Research (SRT) – Course Program 2024

The SRT method is used both for a systematic review and is also relevant when writing background and discussion paragraphs in a PhD or an original article.

DAY 1 (date)
https://deic.zoom.us/j/7337652572 or The Parker Institute, Frederiksberg Hospital

Subject	Time	Form	Lecturer
Welcome<br>Pre test<br>Presentation of Synopses<br>Intro: What is a Systematic Review?	8:30<br><br>9:15	All<br>All<br>Plenary	
Coffee			
Design of a question fit for a Systematic Review<br>Presentation and discussion		Group Teaching<br><br>All	
Lunch	12:30-13:00		
Pit Falls in knowledge search<br><br>Design of a systematic search strategy incl. criteria for inclusion and exclusion etc.		Plenary<br><br>Plenary<br><br>Individual Teaching	
Coffee			
Improvement of own knowledge search<br><br>Perform the search strategy		Plenary<br><br>Individual Teaching	
Sort reg. relevance; Titles, Abstracts and Articles.<br>Trial Profile.	- 16.00	Group Teaching	
**Product of the day: Trial profile with identified articles for the full text evaluation**			

DAY 2 (date)
https://deic.zoom.us/j/7337652572 or The Parker Institute, Frederiksberg Hospital

Subject	Time	Form	Lecturer
Assessment of evidence and quality<br>- RCT study<br>- Cohort/CC-design study	13:00		
Coffee			
Extraction of data	- 16:00		

Practical Course in Systematic Review Technique in Clinical Research (SRT) – Course Program 2024

DAY 3 (date)

https://deic.zoom.us/j/7337652572 or The Parker Institute, Frederiksberg Hospital

Subject	Time	Form	Lecturer
Introduction to Covidence - a tool for streamlined review production	8:30 - 9:30	Plenary	
Coffee			
Introduction to Covidence - a tool for streamlined review production	9:30 - 11:30	Exercise	
Lunch	11:30-12:15		
Clinical & statistical heterogeneity<br>Sub group & sensitivity analyses	12:15	Plenary	
Coffee			
Meta-analysis<br>Quality assessment across the studies (GRADE)	- 16:00	Plenary<br>Exercise	
**Product of the day: Relevant articles from your own search for your review**			

HOME WORK BETWEEN DAY 3 AND 4 (depending on the number of hits in your search)

- **Read the relevant articles for your study, identify their research method and score their evidence- and quality level**
- **Update your synopsis**
- **Write background, material and method paragraphs as well as Trial Profile to own protocol to systematic review**
- **Make to-do list for the rest of your protocol**

DAY 4 – (date)

https://deic.zoom.us/j/7337652572 or The Parker Institute, Frederiksberg Hospital

Subject	Time	Form	Lecturer
Since last time, status and ”Problem Solving”	8:30	Plenary<br>Groups	
Coffee	10:00		
Critical appraisal and evaluation of Systematic Review	10:15-	Plenary	
Lunch	12:30-13:00		
Dissemination and Implementation of results in clinical practice	13:00-15:15	Plenary<br>Groups	
Coffee			
Post test	15:15-15:45	All	
Closure of the course, resume, mutual evaluation	15:45-16:00	All	
**Product of the Day: Own protocol for Systematic Review**			

This programme might be subject to changes
