# Supplementary material for: Learning effect of online versus onsite education in health and medical scholarship – protocol for a cluster randomized trial
Source: BMC Med Educ. 2024 Aug 26;24:927. doi: 10.1186/s12909-024-05915-z (PMC11348670; doi:10.1186/s12909-024-05915-z)
Supplement: Supplementary file 2 — Supplementary Material 2. [file 12909_2024_5915_MOESM2_ESM.pdf]

Course Programme
Getting started: Writing your first manuscript for publication

HOMEWORK BEFORE DAY 1 (expected time approximately two days)

1. Make yourself familiar with the background literature relevant for the article you are working on during the course (your own article).
2. Have an overview of the results you wish to communicate in your article.
3. Choose a scientific journal which will be your first choice and read their author instructions.
4. Ask your supervisor to send you a good scientific article with their comments as to why they think it is a good article (Is it well written? Does it have a clear purpose? Etc.) and send the article and your supervisor's comments to us at whoccuv.bispebjerg-frederiksberg-hospitaler@regionh.dk Deadline xx.
5. Write a brief synopsis about the project you want to write your article about during the course and send it to us at whoccuv.bispebjerg-frederiksberg-hospitaler@regionh.dk Deadline xx.
6. Check if you can enter the course website and read the course material for all course days. Choose the link with the course date and enter with password xx. https://www.clinhpcentre.org/introduktiontilvidenskabeligartikel
7. Set up a Google Scholar Profile https://scholar.google.com, send the link of your profile to whoccuv.bispebjerg-frederiksberg-hospitaler@regionh.dk Deadline xx and identify the following:
    a. How many articles have you published? (All articles count regardless of your position in the author order, but conference abstracts do not count).
    b. If you have published articles: How many times have your articles been cited? (Here everything counts).
    c. If you have been cited: What is your H-index? What is your i10-index?

Summary of preparations with deadline xx

- Send the article and your supervisor's comments
- Send synopsis about the project you want to write about during the course
- Send the link of your Google Scholar profile

Course Programme
Getting started: Writing your first manuscript for publication

DAY 1 (date): FOCUS & PURPOSE
https://deic.zoom.us/j/7337652572 or The Parker Institute, Frederiksberg Hospital

Subject	Time	Form	Lecturer
Welcome & Introduction	8:30		
Pre-test	8:45-9:15	Exercise	
Selected articles:<br>- Focus on the purpose of the article<br>- Composition of the introduction	9:15	Lecture and Exercise<br>Individual reflection and general presentation.	
Coffee			
Selected articles:<br>- Composition of the discussion		Lecture	
Lunch	12:30-13:15		
Individual writing time<br>What works for you as reader and author-to-be? Why?<br>What can you use from this morning's lecture when structuring your own article?	13:15-14:00	Exercise	
What is the purpose of your research?<br>What is the purpose of your article?<br>What is the research question?<br>Tools:<br>Forming focused questions using PICO	14:00-14:45	Lecture	
Coffee	14:45-14:55		
What can you use from the lecture and the discussions regarding the purpose of an article in your own article?<br>Write a draft of the purpose of your article.	14:55-15:55	Exercise/individual reflection and writing<br>Discussion and presentation in groups	
Introduction to homework	15:55-16:00		
**Product of the day: A qualified draft of the purpose of your article**			

Course Programme
Getting started: Writing your first manuscript for publication

HOMEWORK BETWEEN DAYS 1 AND 2 (expected time approximately two hours)

- **First describe your article in four sentences:**
  - **What is the purpose of this specific article?**
  - **How is this purpose part of the overall purpose of your PhD Project?**
  - **Which results do you want to communicate in your article?**
  - **Why is this article relevant to others?**

- **Then describe what your article is about in one core sentence and send it to whoccuv.bispebjerg-frederiksberg-hospitaler@regionh.dk Deadline xx.**

DAY 2 (date): CONTENT & STRUCTURE
https://deic.zoom.us/j/7337652572 or The Parker Institute, Frederiksberg Hospital

Subject	Time	Form	Lecturer
Catch up on challenges from home work	8:30		
Writing methodologies and processes I<br><br>What works for you as an author – why?	9:00	Lecture/exercise Individual reflection and presentation.	
Coffee	10:15-10:30		
Writing methodologies and processes II<br><br>What can you use from the lecture on Writing methodologies and processes in your article?		Lecture/exercise Individual reflection and presentation.	
Lunch	12:00-12:45		
**Content:** What should you include in your article?<br><br>Select what is important and relevant for your article in light of the purpose of your article.	12:45	Lecture<br><br>Exercise. Individual reflection and writing	
Coffee	14:00-14:15		
**Structure:** Dispositions for different types of articles.<br><br>How is each paragraph (introduction, method, results etc.) structured? How do you make sure, that you include what is important for your article?	14:15-15:00	Lecture	
Composition of your article	15:00-15:15	Exercise / begin structuring your article	
Introduction to homework	15:15-15:30		
**Product of the day: Idea of content and qualified draft of the disposition of your article**			

Course Programme
Getting started: Writing your first manuscript for publication

HOMEWORK BETWEEN DAYS 2 AND 3 (expected time approximately one day)

- **With the purpose in mind, write a draft of the background section and polish the disposition of your article.**
- **Pick a sentence you struggle particularly with from your manuscript and which Stephen is allowed to use as an example of how to improve in his lecture and send it to xx. Deadline xx.**
- **Stephen will pick three sentences for his lecture.**
- **Consider in which areas you would like supervision during the individual writing time.**
- **Reflect on a question you would like to ask a journal editor if you had the chance.**

Day 3 (date): WRITING
https://deic.zoom.us/j/7337652572 or The Parker Institute, Frederiksberg Hospital

Subject	Time	Form	Lecturer
Catch up on challenges from home work	8:30		
Academic English	9:00	Lecture	
Coffee	10:15-10:30		
Examples of improvement of sentences<br><br>Supervised individual writing	10:30<br><br>11:00	Lecture<br><br>Exercise/Individual supervision	
Lunch	12:00-12:45		
Individual writing – follow up		Exercise/Individual supervision	
Coffee	14:00-14:15		
Final advice on preparing a manuscript – Tips from an Editor	14:15	Lecture	
Post test	15:00-15:30		
Evaluation and closing	15:30-15:45		
**Product of the day: Qualified draft of an individually selected focus area in your article**			

This programme might be subject to changes
