# Supplementary material for: Learning effect of online versus onsite education in health and medical scholarship – protocol for a cluster randomized trial
Source: BMC Med Educ. 2024 Aug 26;24:927. doi: 10.1186/s12909-024-05915-z (PMC11348670; doi:10.1186/s12909-024-05915-z)
Supplement: Supplementary file 3 — Supplementary Material 3. [file 12909_2024_5915_MOESM3_ESM.pdf]

Appendix x (Information in welcome letter and information and informed consent on first course day

Welcome letter

"At the course we routinely evaluate your experience of the course organization, materials, and lecturers as well as your learning outcome by a pre and post test and your level of motivation and self-efficacy after the course. In a project we also want to follow up on the effect of the course on your scientific activity through the public accessible and broadest covering publication database Google Scholar. […] For more information, please turn to Rie Raffing at whoccuv.bispebjerg-frederiksberg-hospitaler@regionh.dk".

First course day

At the first course day the students are further orally informed about the project details in the introduction by Rie Raffing and given the opportunity to ask questions. Subsequently, they are asked for informed consent in writing in SurveyXact® by the following statement:

"Learning effects of onsite and online research courses

In this course, we run the research project Learning effects of onsite and online research courses with the purpose of investigating the learning effects of onsite and online research courses. We would like to use the data from your evaluation of the course. The data will be anonymized, and it will not be possible to trace any information back to you. Your course and evaluation form will be the same whether you participate in the project or not. You can withdraw your informed consent at any time without any consequences.

Informed consent

□ I give informed consent that my evaluation data from this course can be used in the research

project "Learning effects of onsite and online research courses"

□ I do not give informed consent that my evaluation data from this course can be used in the research project "Learning effects of onsite and online research courses"”
