# Supplementary material for: Learning effect of online versus onsite education in health and medical scholarship – protocol for a cluster randomized trial
Source: BMC Med Educ. 2024 Aug 26;24:927. doi: 10.1186/s12909-024-05915-z (PMC11348670; doi:10.1186/s12909-024-05915-z)
Supplement: Supplementary file 4 — Supplementary Material 4. [file 12909_2024_5915_MOESM4_ESM.pdf]

MCQ questions - 25

1 You have designed a very successful laparoscopy simulation course using state-of-the-art virtual reality simulators. Post-course questionnaires show that all trainees liked the course and believe that it made them better surgeons in the real world. Where should you plan to publish these results?

(1) In a clinical journal that focus on laparoscopy, e.g. Surgical Endoscopy
(2) In a medical education journal that focus on simulation, e.g. Simulation in Healthcare
(3) In your simulation centre's local newsletter or annual report

3

2 What is the most time-consuming part of the research process?

(1) Writing the protocol explaining why and how you plan to do the study
(2) Analysing the data that you have sometimes spent months to gather
(3) Writing the limitations section in a perfect way that can convince the reviewers to accept your manuscript

1

3 What does NOT belong in a good introduction?

(1) A careful description of your local setting explaining the problems that caused you to conduct the study
(2) A general "framing" that very briefly introduces the reader to the field
(3) A research question / an aim of study

1

4 Choose the best research question

(1) What procedures do robotic surgeons think are the most important to include in a simulation-based curriculum?
(2) The Delphi method is a very useful scientific method used to gather expert opinions from key opinion leaders
(3) Why is it very important to use simulation-based training when starting to perform robotic surgery?

1

5 Choose the best research question

(1) Can a 10-hour intensive training course on a virtual-reality laparoscopy simulator compared to no training improve surgical trainees?
(2) Are simulator feedback, expert feedback, and feedback from peers associated with the learning curves of new surgical trainees in laparoscopy?
(3) What is the best way to teach laparoscopic skills to new surgical trainees?

2

6 Choose the best research question

(1) How does the experience of raters influence the reliability of video-based assessment of laparoscopic skills?

(2) Is surgical training on virtual-reality laparoscopic simulators better than training on live pigs?

(3) How do the first-year surgical trainees at Hvidovre Hospital in Copenhagen, Denmark rate their experiences with simulation-based laparoscopy training?

1

7 How should you conduct your statistical analyses?

(1) By manual calculation to become familiar with the formulas

(2) By using a spreadsheet program, e.g. Excel

(3) By using an advanced statistics program, e.g. SPSS

3

8 Choose the best research question

(1) Why is virtual-reality training of laparoscopic skills better than apprenticeship learning?

(2) How many hours should a surgical trainee practice on a virtual-reality simulator before starting to operate on real patients?

(3) Can surgeons returning from a holiday of three weeks or longer benefit from simulation-based ”warm-up” training in terms of reduced time to perform their first post-holiday clinical procedure?

3

9 You have conducted a small scientific study and found negative results. Why should the results be published?

(1) The research ethics committee requires publication of positive, negative and inconclusive results

(2) Your study is the first study on this research question in the world, and even negative results from a small study would be interesting and relevant

(3) Publication is necessary for your PhD and/or your department

1

10 The head of research at your department approves the final version of your manuscript and agrees to be accountable for all aspects of the work. He would like to be co-author. What should you answer him?

(1) “Your support is very valuable and you are welcome to be a co-author because you fulfil two authorship criteria”

(2) “You need to revise the manuscript critically for important intellectual content to fulfil three authorship criteria and thereby qualify to be a co-author”

(3) “You are not qualified to be a co-author because you only fulfil two out of four authorship criteria”

3

11 You are about to begin writing your article and are going to choose where to publish. What do you do?

(1) Read some articles in the journals relevant for your area

(2) Look into your inbox and pick the latest journal invitation to send a manuscript for very fast publication

(3) Draw lots

1

12 You want to do the best possible study comparing a brand new way of caring for post-operative patients with the traditional way. Where should you seek help to prepare your protocol and report your findings?

(1) In the CONSORT Statement

(2) In the PRISMA Statement

(3) In the CARE guidelines

1

13 You need an explicit protocol (before starting) if you are planning to do a

(1) Systematic review

(2) Meta-analysis

(3) Both of the above

3

14 You are planning to systematically go through all clinical trials regarding minimal invasive surgery in third world countries. Where should you register your protocol (before starting)

(1) ClinicalTrials.gov

(2) PROSPERO

(3) WHO.int

2

15 You are about to do a project. Why is it – seen from a scientifically theoretical as well as practical perspective – important to have a good Material & Methods section in your protocol?

(1) The section ensures that you have carefully considered how to collect, store, analyse and publish your results

(2) The section is your ”cook book” for how you want to conduct the project, to later remember what you did, to ensure reproducibility, and to ensure that other researchers can understand what you did

(3) The section is required for publication according to the IMRAD model, which most scientific journals recommend in their instructions to authors

2

16 You are part of a working group which is developing evidence-based guidelines for a new treatment. Which of these designs give the highest evidence level?

(1) Cohort studies
(2) Clinically controlled studies
(3) Case-control studies

2

17 Evidence-based clinical guidelines should describe the following

(1) Evidence, staff competencies and cost-effectiveness
(2) Evidence, patient preferences and cost-effectiveness
(3) Evidence, staff competencies and patient preferences

3

18 Cross-sectional studies are often used because they are quick and cheap. They are characterized by:

(1) Not investigating causal relations
(2) Being at the same evidence level as cohort studies if they are correctly performed
(3) Both of the above

1

19 You are assessing the quality of an RCT and focus among other things on

(1) Inclusion and follow-up rates
(2) Whether the abstract corresponds with the main text of the article
(3) Both of the above

1

20 Patient cases suggest that Covid-19 gives long-term sequelae. You want to investigate patients' challenges with this by using qualitative in-depth interview study, which is characterized by:

(1) Establishing evidence, like a cohort study
(2) Giving insight into and understanding patient experiences
(3) Both of the above

2

21 You are the project leader of a new research project. The Principal Investigator (PI) is eager to get started. The project seems overwhelming and you haven't got a clear picture of the tasks involved and how to prioritize them. What do you do?

(1) Together with the PI, start with the parts that seem the easiest and handle the complexity bit by bit as the project progresses
(2) Set up a meeting with the PI and three senior colleagues who have done similar projects and ask their advice
(3) Discuss with the PI spending time breaking things down in manageable parts, setting deadlines, including buffer time and using a time management tool

3

22 You are about to develop your search strategy, but you are uncertain about how to proceed. What do you choose to base the strategy on?

(1) The hypothesis

(2) The aim

(3) The research question

3

23 A clinical question should be directly relevant to the problem statement. You are eager to phrase your question so that it facilitates a search for a precise answer. What do you do?

(1) Use the PICO format (Patient, Intervention, Comparison, Outcomes)

(2) Identify a question from a similar project and adjust it to fit your research focus

(3) Phrase the question the best you can, perform the search and then adjust the question. Repeat this process until you have phrased a question facilitating a precise answer

1

24 You are planning a systematic review but a relevant pilot-tested search strategy results in a lot of hits. What should you do in such a situation?

(1) Reduce the number of hits by only including publications in English from the last five years

(2) Adjust the research question to better fit the literature and get fewer hits

(3) Accept the number of hits and continue

3

25 Why is it relevant as a researcher to register with Google Scholar?

(1) When put on the internet, my research will automatically be prioritised in Google's search engine

(2) It gives me up-to-date indexes of how much my work is cited

(3) When my work is h-indexed in Google Scholar it will also be indexed in PubMed

2
