# Supplementary material for: Learning effect of online versus onsite education in health and medical scholarship – protocol for a cluster randomized trial
Source: BMC Med Educ. 2024 Aug 26;24:927. doi: 10.1186/s12909-024-05915-z (PMC11348670; doi:10.1186/s12909-024-05915-z)
Supplement: Supplementary file 5 — Supplementary Material 5. [file 12909_2024_5915_MOESM5_ESM.pdf]

Intrinsic Motivation Inventory

1. I believe I had some choice about doing this course

(1) ❍ 1 Not at all true

(2) ❍ 2

(3) ❍ 3

(4) ❍ 4 Somewhat true

(5) ❍ 5

(6) ❍ 6

(7) ❍ 7 Very true

2. I think this is an important course

(1) ❍ 1 Not at all true

(2) ❍ 2

(3) ❍ 3

(4) ❍ 4 Somewhat true

(5) ❍ 5

(6) ❍ 6

(7) ❍ 7 Very true

3. I believe doing this course could be beneficial to me

(1) ❍ 1 Not at all true

(2) ❍ 2

(3) ❍ 3

(4) ❍ 4 Somewhat true

(5) ❍ 5

(6) ❍ 6

(7) ❍ 7 Very true

4. I felt very tense while doing this course

(1) ❍ 1 Not at all true

(2) ❍ 2

(3) ❍ 3

(4) ❍ 4 Somewhat true

(5) ❍ 5

(6) ❍ 6

(7) ❍ 7 Very true

5. I did this course because I wanted to

(1) ❍ 1 Not at all true

(2) ❍ 2

(3) ❍ 3

(4) ❍ 4 Somewhat true

(5) ❍ 5

(6) ❍ 6

(7) ❍ 7 Very true

6. I'd like a chance to interact with the other course participants more often

(1) ❍ 1 Not at all true

(2) ❍ 2

(3) ❍ 3

(4) ❍ 4 Somewhat true

(5) ❍ 5

(6) ❍ 6

(7) ❍ 7 Very true

7. I enjoyed doing this course very much

(1) ❍ 1 Not at all true

(2) ❍ 2

(3) ❍ 3

(4) ❍ 4 Somewhat true

(5) ❍ 5

(6) ❍ 6

(7) ❍ 7 Very true

8. I am satisfied with my performance at this course

(1) ❍ 1 Not at all true

(2) ❍ 2

(3) ❍ 3

(4) ❍ 4 Somewhat true

(5) ❍ 5

(6) ❍ 6

(7) ❍ 7 Very true

9. It is likely that the other course participants and I could become friends if we interacted a lot

(1) ❍ 1 Not at all true

(2) ❍ 2

(3) ❍ 3

(4) ❍ 4 Somewhat true

(5) ❍ 5

(6) ❍ 6

(7) ❍ 7 Very true

10. I felt pressured while doing the course

(1) ❍ 1 Not at all true

(2) ❍ 2

(3) ❍ 3

(4) ❍ 4 Somewhat true

(5) ❍ 5

(6) ❍ 6

(7) ❍ 7 Very true

11. I was pretty skilled at this course

(1) ❍ 1 Not at all true

(2) ❍ 2

(3) ❍ 3

(4) ❍ 4 Somewhat true

(5) ❍ 5

(6) ❍ 6

(7) ❍ 7 Very true

12. I would describe this course as very interesting

(1) ❍ 1 Not at all true

(2) ❍ 2

(3) ❍ 3

(4) ❍ 4 Somewhat true

(5) ❍ 5

(6) ❍ 6

(7) ❍ 7 Very true
