# Supplementary material for: Learning effect of online versus onsite education in health and medical scholarship – protocol for a cluster randomized trial
Source: BMC Med Educ. 2024 Aug 26;24:927. doi: 10.1186/s12909-024-05915-z (PMC11348670; doi:10.1186/s12909-024-05915-z)
Supplement: Supplementary file 6 — Supplementary Material 6. [file 12909_2024_5915_MOESM6_ESM.pdf]

Self-Efficacy

1. I am confident in my ability to solve problems that I might face in life (For example: I can usually handle whatever comes my way, If I try hard enough I can overcome difficult problems, I can stick to my aims and accomplish my goals)

(1) ❍ 1 Strongly disagree

(2) ❍ 2

(3) ❍ 3

(4) ❍ 4

(5) ❍ 5

(6) ❍ 6

(7) ❍ 7

(8) ❍ 8

(9) ❍ 9

(10) ❍ 10 Strongly agree
