# Supplementary material for: Learning effect of online versus onsite education in health and medical scholarship – protocol for a cluster randomized trial
Source: BMC Med Educ. 2024 Aug 26;24:927. doi: 10.1186/s12909-024-05915-z (PMC11348670; doi:10.1186/s12909-024-05915-z)
Supplement: Supplementary file 7 — Supplementary Material 7. [file 12909_2024_5915_MOESM7_ESM.pdf]

If students withdraw from the course after being informed of the setting, a mail is sent to them enquiring of the reason for withdrawal and reason is recorded:

“Dear xx

We have received information that you have declined to participate in the PhD course xx beginning the xx 202x. Please, inform us why you declined to participate in the course. Providing us with this information will help us to improve the course.

Could you please choose one of these reasons for not participating?

1) The course is no longer relevant for me
2) The timing is not possible for me
3) Unexpected practical issues
4) The onsite/online course form is not appropriate for me
5) Other (please elaborate) …

Yours sincerely,

Rie Raffing on behalf of the Course Management”
